# Overexpression of JARID1B is associated with poor prognosis and chemotherapy resistance in epithelial ovarian cancer

**DOI:** 10.1007/s13277-014-2859-z

**Published:** 2015-02-08

**Authors:** Lishuang Wang, Yuanfu Mao, Guiqin Du, Chunbo He, Shiyu Han

**Affiliations:** 1 Department of Gynecology and Obstetrics, The Fourth Affiliated Hospital of Harbin Medical University, Yiyuan Road 37, Nangang District, Harbin 150001 China; 2 Department of Clinical Medicine, Harbin Medical University, Xuefu Road 194, Nangang District, Harbin China; 3 Department of Clinical Laboratory, The Second Affiliated Hospital of Harbin Medical University, Xuefu Road 19, Nangang District, China

**Keywords:** Epithelial ovarian carcinoma (EOC), JARID1B, Prognosis, Chemotherapy resistance

## Abstract

JARID1B, a histone demethylase, has been reported to be highly expressed in various human cancers. In the present study, we investigated the association of JARID1B level with epithelial ovarian cancer (EOC) and prognosis of patients with EOC. We analyzed JARID1B expression in 20 normal ovaries, 20 benign ovarian tumor (BOT) samples, and 45 epithelial ovarian carcinoma specimens by quantitative PCR (qRT-PCR) and western blotting analyses. JARID1B was further examined in 120 EOC specimens from patients with different histological stages via immunohistochemistry. Possible correlations between JARID1B levels and prognosis as well as chemotherapy resistance of EOC patients were determined by univariate and multivariate analyses. JARID1B level was significantly increased in EOC, as compared to normal ovaries and BOT. Among 120 EOC cases examined, the 5-year progression-free survival (PFS) rates were 17 and 85 % in patients with high and low JARID1B expression, respectively (hazard ratio = 17.85, 95 % confidence interval (CI) 6.31–50.51, *P* < 0.001). Similarly, the 5-year overall survival (OS) rates for patients with high and low JARID1B expression were 28 and 92 % respectively (hazard ratio = 21.8, 95 % CI 5.92–71.81, *P* < 0.001). Positive correlation between JARID1B level and chemotherapy resistance was observed in patients with EOC (odds ratio (OR) 36.81, 95 % CI 4.84–280.11, *P* < 0.001). JARID1B could serve as an important biomarker for prognosis and chemotherapy resistance of EOC patients.

## Introduction

Ovarian carcinoma accounts for high lethality in women malignancies worldwide [1]. Of all ovarian cancers, stages I to IV are 7.2, 8.7, 72, and 12.1 % respectively [2]. Due to lacking of effective tools for early detection, most patients with EOC present with advanced stages at diagnosis. The prognosis of EOC is usually poor with a 5-year survival rate around 46 % [3]. Although sophisticated surgery and new chemotherapy regimens appear in the recent years, the 5-year survival rate for patients with EOC was not improved significantly in the last two decades [4]. Failure of EOC treatment is largely due to chemotherapy resistance, an important prognostic index for EOC patients in clinical [3]. Therefore, it is necessary to identify certain effective biomarker that can help to predict the prognosis and chemotherapy resistance of patients with EOC.

JARID1B, also known as PLU1 or KDM5B, is a histone demethylase that converts tri- and di-methylated lysine 4 in histone H3 (H3K4me3/2) to the mono-methylated form (H3K4me1) [5–7]. JARID1B is highly expressed in human cancers of the prostate, lung, and bladder as well as many cancer cell lines [8, 9]. More recently, JARID1B came into the spotlight for its association with a slow-cycling cell population and drug resistance in melanoma [10, 11]. However, the impact of JARID1B expression on the prognosis and chemotherapy resistance of patients with EOC has not been reported yet. Thus, the purpose of this study was to determine whether JARID1B expression level is associated with the prognosis and chemotherapy resistance of EOC and in turn improves the survival rate of EOC patients by regulating JARID1B expression.

## Materials and methods

### Patients and treatment

A total of 120 cases of EOC patients were selected for our study following the approval of the Institutional Review Board, The Fourth Hospital of Harbin Medical University. The criteria for selection include the following: (1) confirmed diagnosis of EOC by pathological examination, (2) complete clinical records, (3) no serious complications or other malignant diseases, (4) absence of prior treatment for cancer, and (5) already had cytoreductive surgery. All selected patients were informed about the illness and consent with the treatment

The selected patients received six courses of intravenous platinum-based combination chemotherapy with 3 weeks between each course. The patients were divided into two groups according to chemotherapy regimens received: (1) TP group (53 patients): paclitaxel (175 mg/m^2^, 1 day) and cisplatin (50 mg/m^2^, 2 days); (2) PAC group (60 patients): cisplatin (70 mg/m^2^), cyclophosphamide (70 mg/m^2^), and epirubicin (50 mg/m^2^).

### Quantitative PCR (qRT-PCR)

Total RNA was extracted from human tissues using the RNeasy Plus Mini Kit (74134, QIAGEN) and then converted into cDNAs using the High Capacity cDNA Reverse Transcription Kit (4368814, Applied Biosystems) following manufacturer’s instruction. Quantitative PCR was performed with a CFX96 (Bio-Rad) using the RT^2^ SYBR Green (330500, SA Biosciences). The primer sequences for JARID1B were as follows: 5′-AGAGGCTGAATG AG CTGGAG-3′ (forward) and 5′-TGGCAATTTTGGTCCATTTT-3′ (reverse). All values were normalized with glyceraldehyde 3-phosphate dehydrogenase (GAPDH) abundance. Data were presented as the average of triplicates ± SD.

### Western blotting analysis

Frozen samples of EOC (45 cases), BOT (20 cases), and normal ovary (20 cases) were homogenized in lysis buffer consisting of 1 % Triton X-100 in phosphate-buffered saline (PBS) supplemented with protease inhibitor cocktail (Roche) and then incubated on ice for 30 min. After that, the mixture was centrifuged at 12,000*g* for 15 min at 4 °C and the supernatant collected. Sixty micrograms of protein extracts were loaded onto 10 % SDS polyacrylamide gel electrophoresis and transferred onto a PVDF membrane (Millipore Company, USA). The blots were blocked with blocking buffer (pH 7.6) containing 5 % nonfat dry milk for 1 h and then incubated with rabbit polyclonal antihuman JARID1B antibody (1:500) (HPA027179, Sigma) overnight at 4 °C. Immunoreactive proteins were stained using a chemiluminescence detection system. Membranes were stripped with stripping buffer for 1 h and re-probed with mouse actin that serves (Neomarkers, Fremont, CA) as an internal control [2, 3].

### Immunohistochemical staining

Paraffin-embedded patient samples of NO (22 cases), BOT (28 cases), and EOC (120 cases) were sectioned onto slides at a thickness of 4 μm. To stain JARID1B, the slides were first deparaffinized in xylene and rehydrated with gradient concentrations of alcohol under standard procedures. After rehydration, the slides were immersed in 0.01 mol/l citrate buffer (pH 6.0) and heated (95 °C) for 15 min for antigen retrieval. Then, the samples were incubated with 3 % hydrogen peroxide (H_2_O_2_) for 10 min followed by 10 % normal goat serum blocking for 10 min. Subsequently, the sections were incubated with primary rabbit polyclonal antihuman JARID1B antibody (dilution 1:100) (HPA027179, Sigma) for 1 h at room temperature. After washing with PBS for 3 times, the sections were incubated with biotin-labeled secondary antibody followed by horseradish peroxidase (HRP)-conjugated streptavidin for 30 min individually at room temperature. After applying HRP substrate, 3.3′-diaminobenzidine tetrahydrochloride (Dako, Germany) in 0.01 % H_2_O_2,_ for 10 min, the slides were counterstained with Meyer’s hematoxylin for 30 to 60 s and mounted with mounting medium for visualization under a microscope.

### Semiquantitative analysis of JARID1B staining

Scoring of JARID1B in EOC samples via immunohistochemistry (IHC) staining follows the methods previously published [2, 3]. All of IHC staining samples from EOC patients were evaluated by two experienced pathologists independently.

### Chemotherapy resistance evaluation

All patients received cytoreductive surgery followed by TP or PAC chemotherapy. Based on the interval from the conclusion of chemotherapy to relapse, the patients were divided into the chemotherapy-sensitive and chemotherapy-resistant groups referring to the standards previously reported [12].

### Follow-up evaluation

During the follow-up period, EOC patients with post-cytoreductive surgery and chemotherapy were requested to perform serum CA-125 test, pelvic MRI, color Doppler ultrasound of the liver and kidney, and pulmonary X-rays every 3 months in the first 2 years, every 6 months from 3 to 5 years, and annually thereafter. The end points of the study were progression-free survival (PFS) and overall survival (OS) as reported [12].

### Statistical analysis

The differences of the demographic characteristics of EOC patients were analyzed by the chi-square (*χ* ^2^) or Fisher’s exact test. The Kaplan-Meier method was used to estimate PFS and OS while the difference in the levels among possible prognostic factors was compared by the log-rank test with univariate analyses. A multivariate Cox regression (proportional hazard model) was employed to identify prognostic factors and evaluate the independent impact of JARID1B level on PFS and OS. The correlation between the level of JARID1B and chemotherapy resistance was assessed by the odds ratio (OR) and the 95 % confidence interval (CI) that were estimated using univariate and multivariate logistic regression with covariate adjustment. Statistical analyses in this study were performed using SAS software (version 9.1.4, SAS Institute, Cary, NC). All statistical tests were two tailed, and *P* values less than 0.05 were considered statistically significant.

## Results

### Demographic characteristics of EOC patients

The demographic and clinical pathological characteristics of EOC patients are listed in Table 1. Of the 120 patients with EOC, the percentage of the International Federation of Gynecology and Obstetrics (FIGO) stages from I to IV is 1.67, 4.17, 90.83, and 3.33 %, respectively. Moreover, there were 110 cases with serum CA-125 level greater than 35 (91.67 %) and 101 cases with ascites volume more than 100 ml. The histopathological types of the EOC included 86 (71.67 %) cases of serous, 14 (11.67 %) cases of mucinous, 18 (15 %) cases of endometrioid, and 2 cases (1 %) of clear cell carcinomas. Lymph node metastasis was observed in 30 patients. Additionally, the distribution range of histopathological differentiation in 120 EOC patients from grades 1 to 3 is 25 (20.83 %), 39 (32.5 %), and 56 (46.67 %), respectively.

**Table 1:** Demographic characteristics of patients with ovarian cancer

Characteristics	JARID1B expression		No. of patients (*N* = 120)	*P*
	Low	High		
Age (years)				0.801
<50	13	30	43	
≥50	22	55	77	
Serum CA-125 level				0.43
<35	4	6	10	
≥35	31	79	110	
Ascites				0.9
<100	6	13	19	
≥100	29	72	101	
Lymph node metastasis				0.0009
Absent	31	49	80	
Present	4	36	40	
Histopathological differentiation^a^				0.0006
G1	13	11	24	
G2	14	26	40	
G3	8	48	56	
Histology type				0.51
Serous adenocarcinoma	27	59	86	
Mucoid adenocarcinoma	6	8	14	
Endometrioid adenocarcinoma	2	16	18	
Clear cell carcinoma	0	2	2	
Residual tumor size				0.11
<1 cm	30	56	86	
1–2 cm	4	18	22	
≥2 cm	1	11	12	
FIGO stage				0.01
I and II	5	3	8	
III and IV	30	82	112	
Chemotherapy regimen				0.025
TP	22	34	56	
PAC	10	52	64	
Chemotherapy resistance				<0.0001
Absent	32	56	88	
Present	0	30	30	

*TP* cisplatin and paclitaxel, *PAC* cisplatin, epirubicin, and cyclophosphamide

^a^G1 is well differentiated, G2 moderately differentiated, and G3 poorly differentiated

### JARID1B expression is increased in EOC

**Fig. 1:**
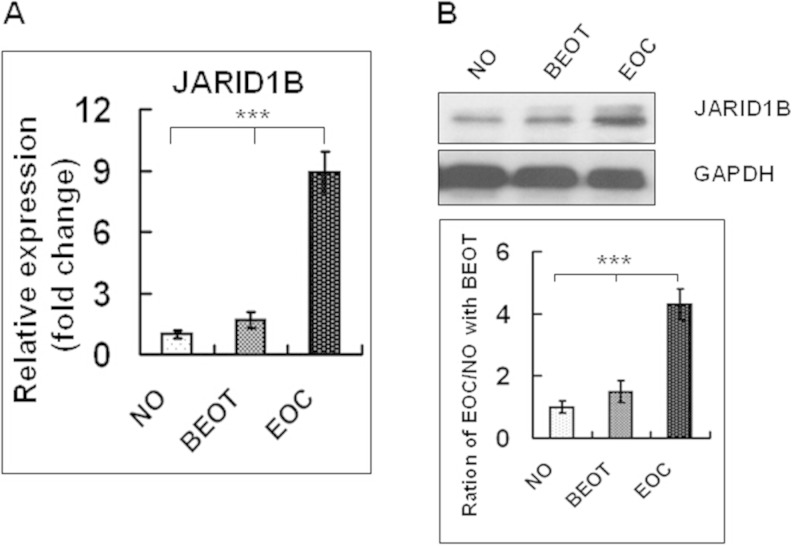
**a** qRT-PCR assay of JARID1B levels in normal ovaries (*NO*), benign ovarian tumor (*BEOT*), and ovarian carcinoma (*EOC*) tissues. GAPDH serves as internal control. **b** The *top panel* presents the Western blotting analysis of JARID1B expression in normal ovaries and in ovarian carcinoma tissues. Protein samples obtained from frozen normal ovaries (*NO*), benign ovarian tumor (*BEOT*), and ovarian carcinomas (*EOC*) were analyzed by SDS-PAGE followed by immunoblotting with antibody against JARID1B. The levels of GAPDH were used as an internal control. The *bottom panel* presents the histogram of pooled data from fresh normal ovaries (*NO*; *n* = 20), benign ovarian tumor (*BEOT*; *n* = 20), and ovarian carcinomas (*EOC*; *n* = 45). The expression of JARID1B was increased both in ovarian carcinomas compared with fresh normal ovaries. Each protein samples was repeated for three times

In the present study, we detected that JARID1B mRNA level was significantly increased in EOC as compared to normal ovary (NO) and benign ovarian tumor (BOT) via qRT-PCR (Fig. 1a). Consistently, the protein level of JARID1B was significantly higher in EOC as compared to NO and BOT (Fig. 1b).

**Fig. 2:**
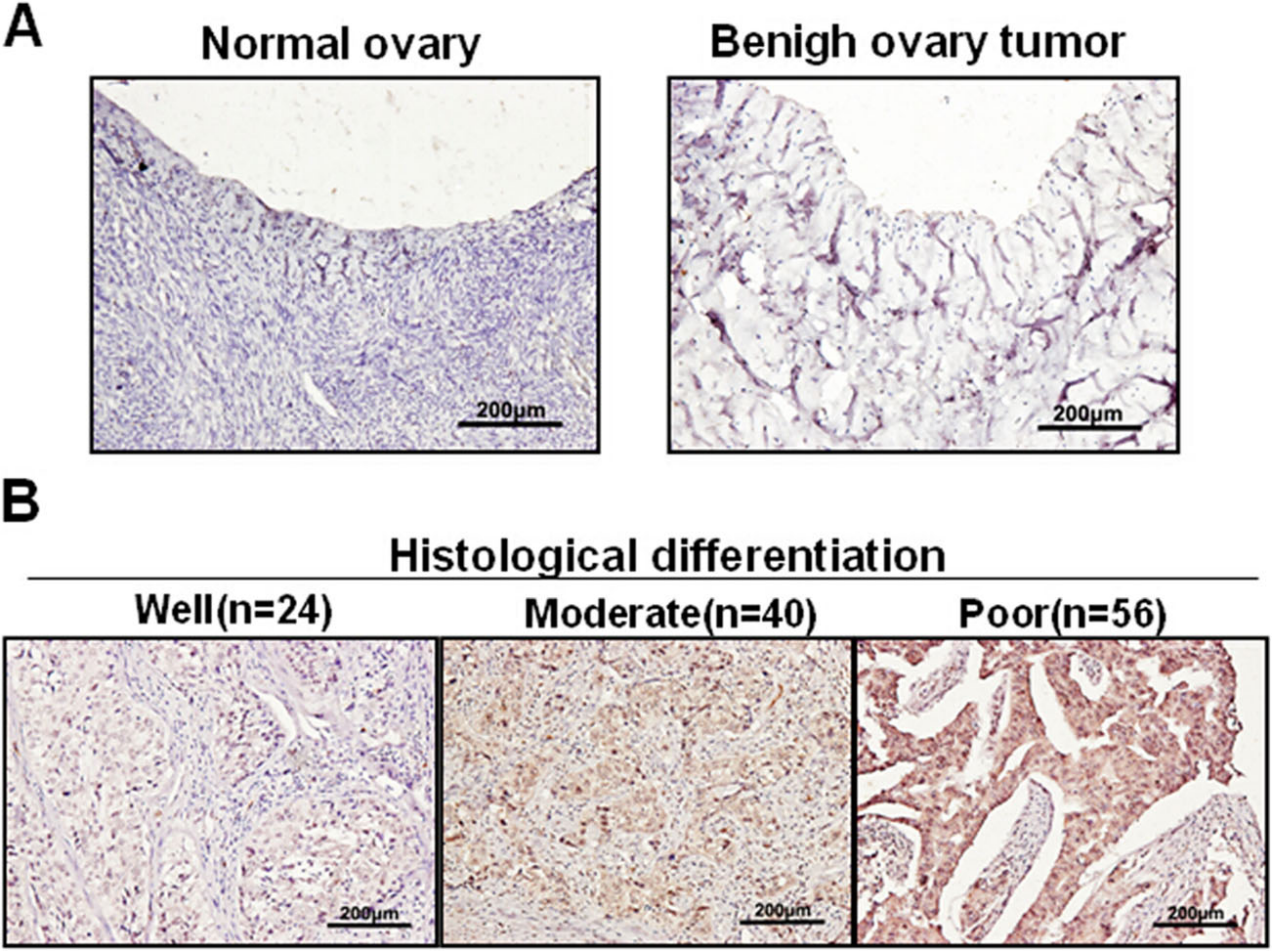
Representative examples show high and low JARID1B expression (positivity + intensity) in tissue samples. **a** The *left panel* shows normal ovaries, score 0 + 0; *right panel* shows benign ovarian tumors, score 0 + 0. **b** The *left panel* shows representative staining patterns of low JARID1B expression (well differentiation), score 1 + 0. The *medium panel* shows representative staining patterns of high JARID1B expression, score: 2 + 1; *right panel* shows representative staining patterns of super-high JARID1B expression, score: 2 + 2. (Original magnification, 200×)

**Fig. 3:**
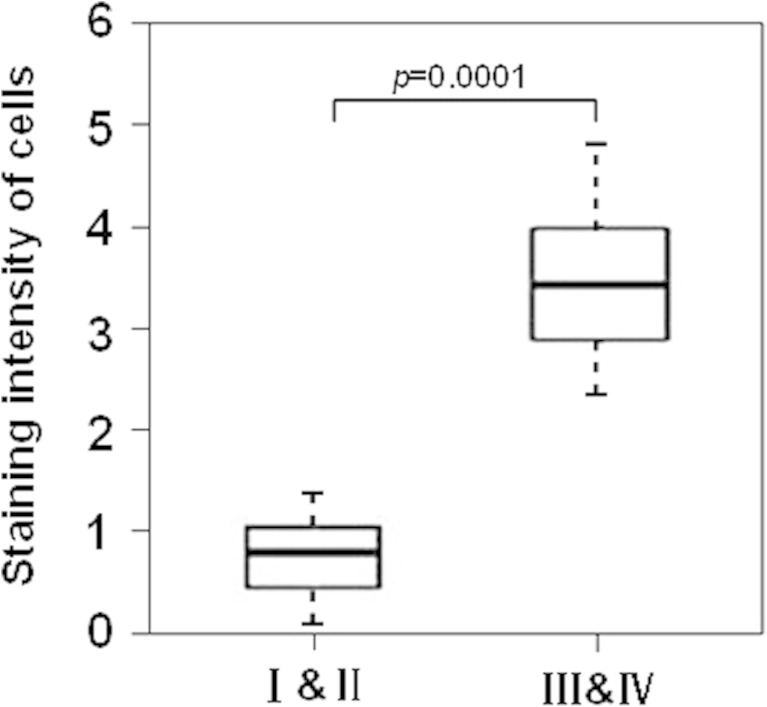
Statistical analysis of JARID1B stain intensity in EOC with FIGO stages (I and II vs III and IV) (*n* = 120, *P* = 0.0001)

Further analyses by immunohistochemical staining revealed that JARID1B was hardly detectable in NO and BOT (Fig. 2a), while strong staining of JARID1B was detected in most EOC samples (Fig. 2b). Interestingly, we noticed that among 120 cases of EOC, 11/24 (45.8 %) of G1, 26/40 (65 %) of G2, and 48/56 (85.7 %) of G3 tumors have a relatively high level of JARID1B (Fig. 2b), suggesting JARID1B level may negatively correlate to EOC histopathological differentiation. Furthermore, a significant difference in lymph node metastasis was observed between JARID1B high- (36/85) and low-expression groups (4/35) (Table 1, *P* < 0.0009). In addition, JARID1B level was significantly higher in late stages (III and IV) than in early stages (I and II) of EOC samples (Fig. 3).

### The association of JARID1B with prognosis of EOC

**Fig. 4:**
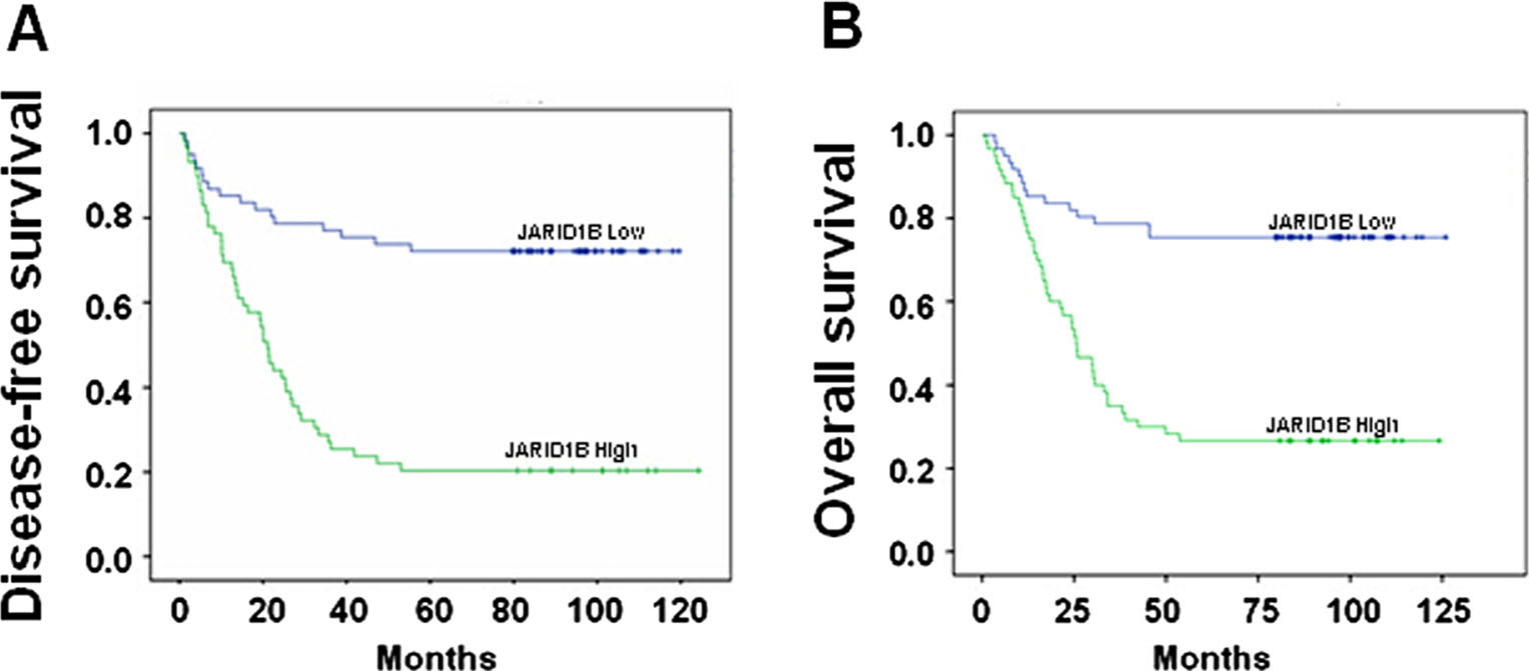
Kaplan-Meier curves for survival of prognosis in 120 patients with epithelial ovarian carcinoma according to the categories of low and high expression of JARID1B (analyzed with log-rank test). **a** Progression-free survival. **b** Overall survival

The Kaplan-Meier curves of PFS and OS for EOC patients with either high or low level of JARID1B were shown in Fig. 4. The average PFS for patients with high expression of JARID1B was 2.37 years, which was significantly lower than that for those with low JARID1B (4.27 years, *P* < 0.001) (Table 2). Similarly, OS for EOC patients with high level of JARID1B was also significantly lower than that for those with low JARID1B expression (*P* < 0.001), which were 4.71 and 3.35 years, respectively (Table 2).

**Table 2:** Univariate analysis of 120 patients with ovarian cancer

Variables	OS		*P*	PFS		*P*
	Mean ± SE	5-year (%)		Mean ± SE	5-year (%)	
JARID1B expression			<.001			<.001
Low	4.71 ± 0.07	92		4.27 ± 0.09	85	
High	3.35 ± 0.24	28		2.37 ± 0.22	17	
Residual tumor size			0.01			0.02
<1 cm	4.45 ± 0.24	51		3.45 ± 0.23	41	
1–2 cm	3.37 ± 0.38	35		2.30 ± 0.33	25	
≥2 cm	1.35 ± 0.29	24		0.60 ± 0.13	22	
Serum CA-125 level			0.16			0.28
<35	3.24 ± 0.37	71		2.07 ± 0.17	57	
≥35	4.07 ± 0.22	44		3.12 ± 0.21	36	
Ascites			0.77			0.96
<100	2.49 ± 0.27	50		2.45 ± 0.37	37.5	
≥100	4.15 ± 0.23	45		3.19 ± 0.22	37	
Lymph node metastasis			0.02			0.003
Absent	3.67 ± 0.21	54		2.86 ± 0.19	48	
Present	3.75 ± 0.33	28		2.53 ± 0.29	14	
Histopathological differentiation^a^			0.12			0.035
G1	3.96 ± 0.34	60		2.09 ± 0.22	52	
G2	3.82 ± 0.29	50		3.13 ± 0.25	44	
G3	3.72 ± 0.32	35		2.65 ± 0.28	26	
Histology type			0.12			0.36
Serous adenocarcinoma	4.35 ± 0.24	48		3.34 ± 0.23	39	
Mucoid adenocarcinoma	3.90 ± 0.49	50		2.01 ± 0.31	41	
Endometrioid adenocarcinoma	2.11 ± 0.35	31		1.41 ± 0.22	26	
Clear cell carcinoma	2.02	100		1.66	100	
Age (years)			0.93			0.91
<50	3.75 ± 0.25	42		2.64 ± 0.23	34	
≥50	4.08 ± 0.28	47		3.19 ± 0.26	39	
Chemotherapy regimen			<.001			<.001
TP	5.33 ± 0.26	73		4.29 ± 0.26	61	
PAC	2.76 ± 0.20	21		2.08 ± 0.21	16	
FIGO stage			0.02			0.004
I and II	4.21478 ± 0.21885	47.46		3.25888 ± 0.20959	38.73	
III and IV	2.28819 ± 0.63153	–		1.08681 ± 0.31846	–	

*OS* overall survival time. *PFS* progression-free survival time, *TP* cisplatin and paclitaxel, *PAC* cisplatin, epirubicin, and cyclophosphamide

^a^G1 is well differentiated, G2 moderately differentiated, and G3 poorly differentiated

Besides JARID1B, we noticed there were some other factors that also had significant impact on PFS and OS of EOC patients in univariate analyses. They include residual tumor size, histopathological differentiation, lymph node metastases, FIGO stage, and chemotherapy regimen (Table 2).

To evaluate the independent impact of JARID1B on the PFS and OS for EOC patients, a multivariate Cox regression model adjusted with other statistically significant prognostic factors described above was performed. As shown in Table 3, EOC patients with high level of JARID1B has a significantly shorter PFS and OS than those with relatively low level of JARID1B (PFS: hazard ratio = 17.9, *P* < 0.001; OS: hazard ratio = 21.8, *P* < 0.001).

**Table 3:** Multivariate analysis with covariates adjustment of 120 patients with ovarian cancer

Prognostic variables	OS^a^			PFS^b^		
	HR	95 % CI	*P* value	HR	95 % CI	*P* value
JARID1B expression						
Low						
High	21.8	5.92,71.81	<.001	17.85	6.31,50.51	<.001
Lymph node metastasis						
Absent						
Present	0.772	0.44,1.35	0.36	0.93	0.55,1.59	0.81
Histopathological differentiation^c^						
G3						
G2	0.94	0.51,1.7	0.84	0.80	0.45,1.41	0.43
G1	1.55	0.69,3.46	0.28	2.27	1.06,4.88	0.04
Chemotherapy regimen						
TP						
PAC	5.14	2.77,9.5	<.001	4.53	2.58,7.96	<.001
Age (years)						
<50						
≥50	1.27	0.72,2.2	0.41	1.16	0.70,1.93	0.56
FIGO stage						
I and II						
III and IV	2.26	0.74,6.90	0.15	5.30	1.71,16.41	0.0038
Residual tumor size						
<1 cm						
1–2 cm	0.79	0.42,1.51	0.48	0.92	0.50,1.71	0.80
≥2 cm	4.078	1.7,9.80	<.001	3.31	1.41,7.74	<.001

*OS* overall survival time, *PFS* progression-free survival time, *TP* cisplatin and paclitaxel, *PAC* cisplatin, epirubicin, and cyclophosphamide

^a^Likelihood ratio test, *P* < .001

^b^Likelihood ratio test, *P* < .001

^c^G1 is well differentiated, G2 moderately differentiated, and G3 poorly differentiated

### Impact of JARID1B overexpression on chemotherapy resistance in ovarian cancers

Among 118 EOC patients who underwent chemotherapy (exclude 2 patients of stage I), 88 patients were chemotherapy sensitive while the other 30 patients presented with chemotherapy resistance, in which 29 (96.67 %) patients expressed high level of JARID1B (Table 4). In addition to JARID1B, chemotherapy resistance was also significantly associated with residual tumor size and chemotherapy regimen (*P* = 0.004 and *P* < 0.001, respectively) (Table 4). However, no significant difference was found between the chemotherapy-sensitive and chemotherapy-resistant groups in other factors such as age, serum CA-125 concentration, ascites, lymph node metastases, histopathological differentiation, and histopathological type.

**Table 4:** Demographic characteristics of patients undergoing chemotherapy with ovarian cancer

Characteristics	Chemotherapy resistance		No. of patients (*N* = 118)	*P* value
	Absent	Present		
Age (years)				0.24
<50	34	8	42	
≥50	54	22	76	
Serum CA-125 level				0.20
<35	8	1	9	
≥35	80	29	109	
Ascites				0.9
<100	13	5	18	
≥100	75	25	100	
JARID1B expression				<0.001
Low	34	1	35	
High	54	29	83	
Lymph node metastasis				0.85
Absent	60	19	79	
Present	28	11	39	
Histopathological differentiation^a^				0.06
G1	19	4	23	
G2	33	6	39	
G3	36	20	56	
Histology type				0.77
Serous adenocarcinoma	64	21	85	
Mucoid adenocarcinoma	11	3	14	
Endometrioid adenocarcinoma	12	6	18	
Clear cell carcinoma	1	0	1	
Residual tumor size				0.004
<1 cm	70	16	86	
1–2 cm	15	8	22	
≥2 cm	3	7	10	
Chemotherapy regimen				<0.001
TP	50	5	55	
PAC	38	27	63	
FIGO stage				0.91
II	2	3	5	
III	83	26	109	
IV	3	1	4	

*TP* cisplatin and paclitaxel, *PAC* cisplatin, epirubicin, and cyclophosphamide

^a^G1 is well differentiated, *G2* moderately differentiated, and *G3* poorly differentiated

To evaluate the independent impact of JARID1B on chemotherapy resistance, a multivariate logistic regression model adjusted with other statistically-significant factor including residue tumor size and chemotherapy regimen was applied. The results revealed that high level of JARID1B was independently associated with chemotherapy resistance (OR 36.81, 95 % CI 4.84–280.11, *P* < 0.001) (Table 5).

**Table 5:** Multivariate analysis of the association between chemotherapy resistance and JARID1B expression in epithelial ovarian cancers

Variables		$\widehat{\beta}$	$SE\left(\widehat{\beta}\right)$	*χ* ^2^	*P* value	OR (95 %CI)
Residual tumor size	<1 cm					
	1–2 cm	0.95	0.58	3	0.12	3.1 (0.95,4.16)
	≥2 cm	1.2	0.68	3.27	0.07	3.6 (1.05,7.43)
Chemotherapy regimen	TP					
	PAC	0.88	0.52	2.90	0.09	2.412 (0.88,6.65)
JARID1B expression	Low					
	High	3.60	1.04	11.91	<0.001	36.81 (4.84,280.11)

$\widehat{\beta}$ and $SE\left(\widehat{\beta}\right)$are the parameter estimator of association coefficient and its standard error

*χ* ^2^ chi-square statistic, *OR* odds ratio, *CI* confidence interval, *TP* cisplatin and paclitaxel, *PAC* cisplatin, epirubicin, and cyclophosphamide

## Discussion

To date, the associations between JARID1B level and prognosis as well as chemotherapy resistance in EOC have not been reported. This is the first study to investigate the impact of JARID1B overexpression on prognosis and chemotherapy resistance using a large number of clinical samples. In this study, we analyzed JARID1B expression in 120 patients with EOC by immunohistochemistry and found that JARID1B was an independent factor for both prognosis and chemotherapy resistance of EOC.

This study analyzed the association between JARID1B expression and traditional clinicopathogical characteristics in EOC. JARID1B expression was associated with lymph node metastases, FIGO stages, and histopathological differentiation, which could be helpful in understanding the progression and prognosis of patients with EOC.

The present data demonstrated that JARID1B overexpression was associated with poor survival by analyzing the OS and PFS. In this study, a multivariate Cox regression analysis indicated that JARID1B expression and histopathological differentiation were independent prognostic factors for OS and PFS in EOC. The results of this study are consistent with previously reported results. Shoji et al. [13] found that high luminal JARID1B activity is associated with poor outcome in patients with hormone receptor-positive breast tumors. Yoshihiro et al. [14] results indicated that JARID1B plays a role in maintaining cancer stem cells in the esophagus and justifies the rationale for studying the effects of continuous inhibition of this epigenetic factor in esophageal cancer. Zhang et al. [15] findings suggest that Myc-mediated transcriptional repression of JARID1B counterintuitively inhibits Myc-regulated cell proliferation and potentially tumorigenesis. Radberger et al. reported a statistically significant association (*P* < 0.05) between JARID1B expression and OS in uveal melanoma [16]. We found similar association in EOC patients. Among 120 EOC samples examined, JARID1B was highly expressed in 85 cases (71 %), suggesting JARID1B might be an independent biomarker for EOC. It has been reported that JARID1B might play an important role in the initiation and development of prostate cancer [17]. In the present study, we observed that JARID1B level was pretty low or nearly undetectable in normal ovary and benign ovarian tumor samples while it was significantly increased in EOC, suggesting JARID1B might also play an important role in tumorigenesis of EOC.

A common reason for poor prognosis of EOC results from chemotherapy resistance. High expression of JARID1B has been reported in a variety of cancers including prostate, melanoma, and breast cancer [6, 11, 17]. Our study is the first to describe the association between JARID1B and chemotherapy resistance as well as prognosis for EOC. Specifically, we found that high level of JARID1B is tightly associated with chemotherapy resistance after adjusted with other factors that correlate to chemotherapy resistance such as residual tumor size and chemotherapy regimen via multivariate logistic regression analysis (OR 36.81, 95 % CI 4.84–280.11, *P* < 0.001), indicating JARID1B is an independent factor for chemotherapy resistance in EOC.

Our study also has some limitations. The sample size is relatively small. Moreover, detailed mechanisms for JARID1B affecting EOC chemotherapy resistance and prognosis remain unclear and require further study.

In conclusion, JARID1B may serve as an important biomarker to predict chemotherapy resistance and prognosis for EOC patients.
